# Supplementation in mushroom crops and its impact on yield and quality

**DOI:** 10.1186/s13568-018-0678-0

**Published:** 2018-09-18

**Authors:** Jaime Carrasco, Diego C. Zied, Jose E. Pardo, Gail M. Preston, Arturo Pardo-Giménez

**Affiliations:** 1 Department of Plant Sciences, University of Oxford, S Parks Rd, Oxford, OX1 3RB UK; 2 Universidade Estadual Paulista (UNESP), Câmpus de Dracena, Dracena, São Paulo 17900-000 Brazil; 3 Escuela Técnica Superior de Ingenieros Agrónomos y de Montes (ETSIAM), Universidad de Castilla-La Mancha, Campus Universitario, s/n, 02071 Albacete, Spain; 4 Centro de Investigación, Experimentación y Servicios del Champiñón, Quintanar del Rey, Cuenca Spain

**Keywords:** Substrate, Mushroom cultivation, Nutrition, Agronomy, Yield, Quality

## Abstract

Mushroom supplementation is an agronomic process which consists of the application of nutritional amendments to the substrates employed for mushroom cultivation. Different nitrogen and carbohydrate rich supplements have been evaluated in crops with a substantial impact on mushroom yield and quality; however, there is still controversy regarding the nutritional requirements of mushrooms and the necessity for the development of new commercial additives. The addition of external nutrients increases the productivity of some low-yielding mushroom varieties, and therefore is a useful tool for the industry to introduce new commercially viable varieties. Spent mushroom compost is a waste material that could feasibly be recycled as a substrate to support a new commercially viable crop cycle when amended with supplements. On the other hand, a new line of research based on the use of mushroom growth promoting microorganisms is rising above the horizon to supplement the native microbiota, which appears to cover nutritional deficiencies. Several supplements employed for the cultivated mushrooms and their agronomic potential in terms of yield and quality are reviewed in this paper as a useful guide to evaluate the nutritional requirements of the crop and to design new formulas for commercial supplementation.

## Introduction

Most of the cultivated species of mushrooms belong to the phylum *Basidiomycota*, although some *Ascomycota* such as members from the genera *Morchella* or *Tuber* have also been successfully cultivated and commercially exploited (Rubini et al. 2014; Liu et al. 2017). Unlike plants, mushrooms are heterotrophic organisms which require external nutrients to grow; the vegetative mycelium (hypha network) supplies nutrients for the growth of basidiomes (reproductive stage) (Taylor and Ellison 2010). Mushrooms produce a number of enzymes including lignin-degrading enzymes (laccases, lignin peroxidases, manganese peroxidases, arylalcohol oxidase, aryl-alcohol dehydrogenases or quinone reductases), and hemicellulose and cellulose-degrading enzymes (xylanase, cellulases or cellobiose dehydrogenase), to facilitate the degradation of lignocellulosic substrates (Sánchez 2009; Kabel et al. 2017; Vos et al. 2017). Furthermore, mushrooms require oxygen and a specific pH in order to develop a normal metabolism and to grow properly. C and N are the two main macronutrients required by fungi for structural and energy requirements; P, K and Mg are also considered macronutrients for mushrooms, in addition, trace elements such as Fe, Se, Zn, Mn, Cu and Mo appear to be needed for diverse functions (Chang and Miles 2004).

The initial phase of mushroom production consists of a solid fermentation process. From spawning, the vegetative mycelium grows under controlled environment and aseptic conditions to colonize the mass of substrate before fructifying (Zervakis and Koutrotsios 2017). There are two main formulas for the production of the substrates employed in mushroom cultivation that have been optimized depending on the species. Both are derived from agricultural by-products such as cereal straw, plant fiber/husk, manure or sawdust:

1. Composted materials achieved through fermentation and pasteurization (Pardo et al. 2017; Kabel et al. 2017; Vos et al. 2017), designed for the cultivation of *Agaricus bisporus* (Lange) Imbach (AB) or *A. subrufescens* Peck (Pardo-Giménez et al. 2014; Pardo et al. 2017), *Pleurotus ostreatus* (Jacq: Fries) (PO), *P. sajor*-*caju* (Fr.) Singer or *P. cistidiosus* O.K. Mill. (Chang and Miles 2004; Sánchez 2010).
2. Non-composted materials that consist of a mixture of different agricultural by-products as the main ingredients, followed by steam sterilization of the substrate prior to the inoculation of the mycelium. Certain commercial species are produced employing this kind of substrate, including *Lentinula edodes* (Berk.) Pegler (LE), *Auricularia* sp., *Flammulina velutipes* (Curtis) Singer, *Pleurotus eryngii* (DC.: Fr.) Quel., *Agrocybe aegerita* (V. Brig.) Singer, *Volvariella volvacea* (Bull. Ex Fr.) or *Hypsizygus marmoreus* (Peck) Bigel (Chang and Miles 2004; Estrada et al. 2009; Liang et al. 2016; Xie et al. 2017; Kleofas et al. 2014; Yamanaka 2017).

Some cultivated species, like the globally cultivated white button mushroom (*A. bisporus*), require a casing overlay to cover the colonized substrate in order to induce mushroom fructification (Pardo-Giménez et al. 2017a).

Mushroom supplementation is understood as a farming method based on the physical addition of nutritional amendments to compost, during the process of composting, the mixture of raw materials, at spawning or during casing (Estrada et al. 2009; Pardo-Giménez et al. 2012a, 2016). The practice of nutritionally supplementing compost for mushroom cultivation at the time of spawning or casing to maximize crop yield emerged in the 1960s (Schisler and Sinden 1962; Sinden and Schisler 1962; Lemke 1963) and is widely recognized and accepted, however its use can be restricted in some sectors because of technical and economic factors. Important aspects to be considered include, on the one hand, the types of nutrients required and the most suitable time for them to be applied without forgetting, on the other hand, economic costs and profits (Randle 1985).

Recently, potential mushroom growth promoting (MGP) fungi and bacteria have been described to stimulate the mycelium growth and promote mushroom fructification, while constituting nitrogen or vitamins reservoirs (Zarenejad et al. 2012; Kertesz and Thai 2018). MGP therefore represent an additional form of supplement that could be supplied separately, or in combination with nutritional supplements, to increase crop yield.

The present mini-review compiles the recent advances on the supplementation of substrates employed in mushroom cultivation and aims to shed light on some agronomical aspects regarding this expanding crop (Zhang et al. 2014).

## Formulation of nutritional additives for mushroom cultivation

Each mushroom species requires an optimal C/N ratio in the substrate employed for cultivation, that allows growers to achieve the highest yield in the shortest period of production (Zied et al. 2011). Supplements are commonly manufactured products containing defatted vegetable meal, such as soybean meal, and other organic protein sources, among them cereal bran, enriched with minerals or vitamins, which are frequently used for the cultivation of *Agaricus* and *Pleurotus* species (Zied et al. 2011; Burton et al. 2015). There are a number of commercial supplements available for the producer in the market, most of them designed to supplement the phase II (at spawning) and phase III (at casing) substrate produced for the cultivation of *A. bisporus*. The commercial products mostly employed are produced by Amycel (Promycel^®^, Titanium^®^, Ultimate^®^, etc.); Champfood (Champfood^®^); Lambert (Full House^®^), Havens (MCSubstradd^®^), Superchamp (Mix P^®^or Mix V^®^), Nutrigain (Nutrigain Organic Gold^®^ or Nutrigain MycroLiquid^®^) and Everris (Micromax^®^), and are based on protein, lipid/protein blends, carboxylic acids or minerals (Burton et al. 2015).

In addition to these existing supplements, the use of low-cost agricultural by-products available at the productive regions is a promising approach. Among others, cereal meals and brans, chicken manure, cottonseed meal, urea, superphosphate, ammonium sulphate, grape pomace, feather flour or defatted meals from dry nuts, are recognized as active ingredients to supplement substrates employed in mushroom cultivation in Brazil or Europe (Zied et al. 2011; Pardo-Giménez et al. 2016, 2018). Following we summarize some of the most relevant results reported while employing agricultural wastes for mushroom supplementation:

Cultivation experiments on substrates supplemented with 20–40% composted or 20% raw two-phase olive mill waste (“alperujo”) revealed a great potential for the cultivation of *Pleurotus* spp. and *Agrocybe cylindracea* while valorizing environmentally hazardous agricultural waste in Greece (Zervakis et al. 2013). *Flammulina velutipes* has been also cultivated in substrates with a high amount of alperujo, resulting in good biological efficiencies while minimizing the highly phytotoxic properties of this contaminating by-product (Rugolo et al. 2016).

Substrates based on grapeseed meal, defatted pistachio meal or defatted almond meal have shown similar agronomical behaviour to commercial additives for the cultivation of *A. bisporus* and *P. ostreatus* in Spain (Pardo-Giménez et al. 2012a, 2016, 2018). Supplement formula consisting of 25% of soybean, black bean, wheat bran and chia also showed good agronomical performance in substrates generated by self-heating pasteurization for the cultivation of *A. bisporus* (Colmenares-Cruz et al. 2017).

Trials conducted adding corn husk, oat husk, soy bean nuggets and peanut shell, in combination with soya as a nutritional supplement, to oyster mushrooms in straw-based substrates at spawning were reported to generate mushrooms with higher protein content (Jeyanthi Rebecca et al. 2015). Other local agricultural materials have been successfully employed as supplements in Iran for the cultivation of oyster mushroom, including wood chips, boll, sugar beet pellet pulp and palm fiber along with wheat bran, rice bran, soya cake powder, soya cake powder and rice bran and carrot pulp (Jafarpour et al. 2010).

## Application of mushroom supplements

The correct timing and methods of application of supplements is an essential condition for obtaining the expected results, with several important culture aspects, such as the composting process, the control of temperature for mycelial growth before and after casing, the hygiene measures, the choice of supplement and its application time and, especially, the uniform distribution of the product used in the substrate, all affecting subsequent yields (Desrumaux et al. 1999).

To achieve a successful supplementation, particularly at spawning, it is necessary to design supplements which retard the availability of nutrients. The delayed-released nutrients where firstly reported by Carrol and Schisler (1976) through a treatment with formaldehyde. According to the authors formaldehyde limited the solubility and denatured the proteins of supplements, in addition it inhibited their availability for competitors moulds and then the mushroom mycelium could take the gradually accessible nutritional content when it became dominant within the mass of compost.

The increase in temperature immediately after supplementing should also be controlled. For instance, in modern facilities (equipped with air-conditioned growing-rooms and mechanized shelves to manage the crop) supplements can be added to the colonized compost just before applying the casing layer. Therefore, because environmental conditions can be controlled, excessive compost temperatures are avoided during spawn running, and the incidence of fungal competitors is minimized.

This also applies when the compost is incubated in tunnels at the composting plant (phase III). However, cropping in bags with phase II compost, the primary system used in many parts of the world, requires supplementation during spawning primarily due to mechanical restrictions (Pardo-Giménez et al. 2017b).

**Fig. 1:**
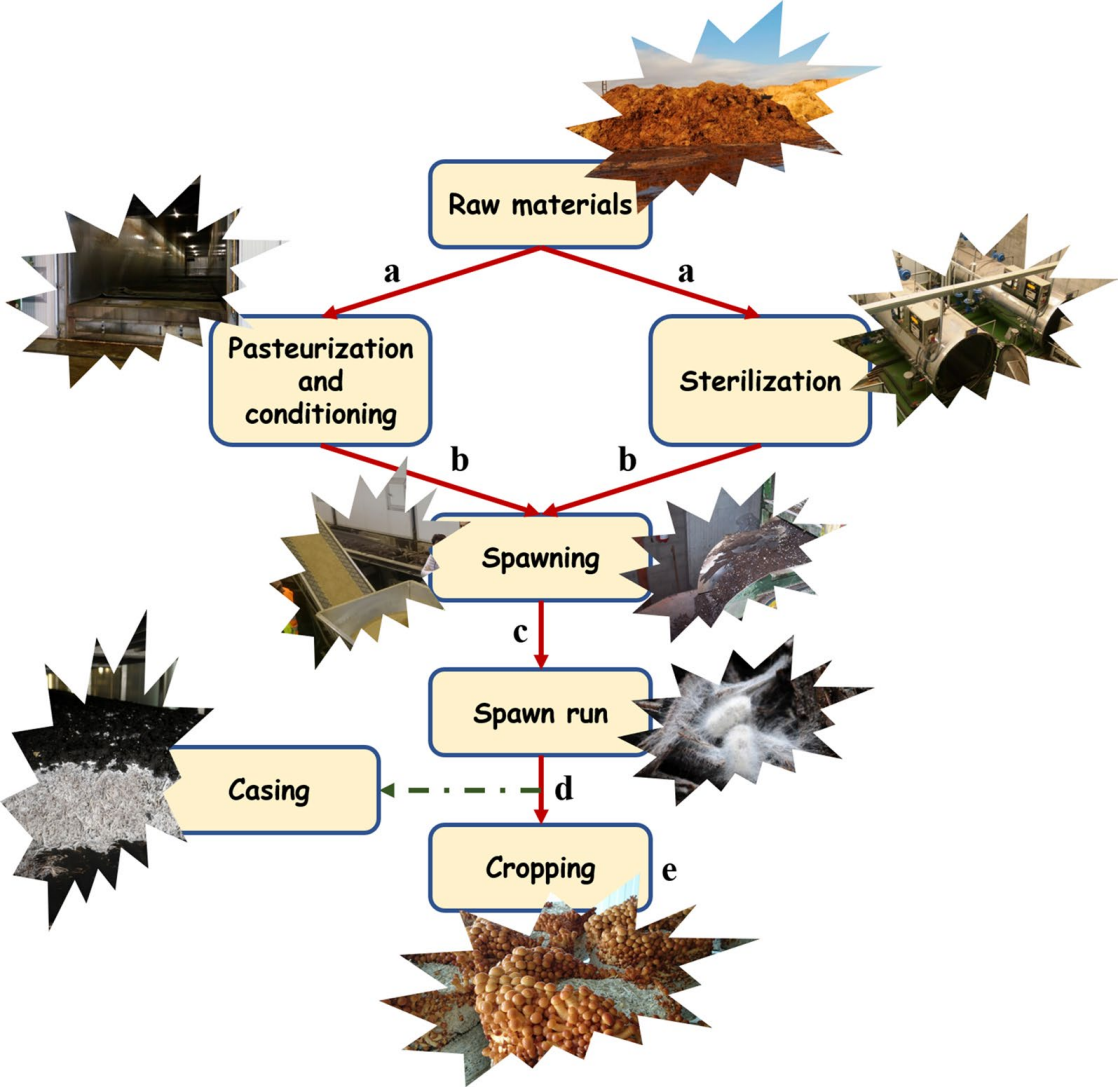
Nutritional supplementation of substrates along different stages of mushroom cultivation. **a** Application to raw materials; **b**, **c** Addition during spawning (after pasteurization/sterilization of substrates); **d** Supplementation of colonized substrates; **e** Supplementation during the cropping stage

Therefore supplements, which are based on slow nutrient release formulas, can be applied at different points along the mushroom cropping cycle (Fig. 1). They are most commonly applied at the end of the substrate preparation, prior to spawning, to promote the vegetative growth throughout the substrate (Naraian et al. 2009), at the end of spawn run (with the substrate fully colonized by mushroom mycelium) to promote the mushroom colonization in the casing material (if required) and enhance mushroom fructification (Pardo-Giménez et al. 2016, 2018). However, the application of supplements along cropping has been also tested to increase the production of late flushes by supplementing the 2nd flush compost in *A. bisporus* (Royse and Chalupa 2009; Royse 2010).

## The nutritional content of mushrooms: effect of supplementation

Cultivated mushrooms are a highly nutritious food that can be grown on biological wastes, agricultural wastes or agro-industrial wastes (Sánchez 2010; Atila 2017). Researchers have reported variations in the nutritional content of mushrooms cultivated on different substrates. When comparing the effects of different agro-wastes on the nutritional composition of oyster mushrooms and *Pleurotus cystidiosus,* formulas with 100% sugar bagasse and 100% corncob showed higher values of protein, fiber, ash and mineral content (Ca, K, and Mg) than 100% sawdust (Hoa et al. 2015). The cultivation of *Pleurotus ostreatus* on various sawdust substrates was reported to give the best nutritional composition in mushrooms for the substrates based on fig tree sawdust, with the mushroom showing the highest amount of dry matter, lipid, nitrogen, iron, zinc and selenium; followed by a mixture of various sawdusts and an Ipil-ipil tree sawdust, all supplemented with 30% wheat bran and 1% lime (Bhattacharjya et al. 2015).

Furthermore, supplementation of substrates in *A. bisporus* with trace elements has been described as reliable for the production of fruiting bodies enriched with Se, Cu and Zn (Werner and Beelman 2002; Rzymski et al. 2017), micronutrients that frequently are deficient in the human diet (Bird et al. 2017).

## Implications on yield and quality

The formula employed for the design of the substrate deeply influences the yield and quality of the basidiomes harvested (Moonmoon et al. 2011; He et al. 2018).

Compost supplementation with defatted pistachio meal and defatted almond meal significantly improved the quality of white button mushroom, *A. bisporus,* (larger mushrooms with firmer texture and greater content in dry weight and protein) and increased more than 30% the yield in oyster mushroom, *P. ostreatus*, in comparison to non-supplemented substrates (Pardo-Giménez et al. 2016, 2018). Sawdust supplemented with different levels of wheat bran, rice bran or maize powder improved yield and quality of *Lentinula edodes*, with 25% wheat bran and 40% wheat bran reported as the best rate to obtained highest yield and best quality respectively (Moonmoon et al. 2011). Among four different formulas evaluated, the addition of *Morchella* spp. footing soil had the optimal effect on promoting the growth and quality of *Morchella* spp. among four different formulas (He et al. 2018). Finally, *Pleurotus* species produced on substrates containing grape marc or olive mill wastes showed higher content of bioactive compounds and comparable productivity than wheat-based substrates (Koutrotsios et al. 2018).

## Supplementation of spent mushroom compost as a recycling material for the cultivation of mushroom species

The annual production of spent mushroom substrate (SMS), the agricultural waste derived from mushroom production, is estimated around 170–204 billion kg in 2013 (Ma et al. 2014; Royse et al. 2017). Among the different uses described for spent mushroom compost (Rinker 2017), the recycling of the SMS through amendment with nutritional supplements to support further mushroom production is a viable alternative to cope with the high volume of this waste material (Pardo-Giménez et al. 2011, 2012b). When the SMS from *P. ostreatus* is supplemented with a protein-rich product, such as commercial supplements (Calprozime^®^, Champfood^®^ or Promycel 600^®^), the total nitrogen content in the substrate increases, and consequently, the values of the crude protein content and the neutral detergent-soluble fraction (Picornell-Buendía et al. 2016a). Substrate formulations with material based on wheat straw and spent *Pleurotus* substrates supplemented with wheat bran and the commercial supplement (Calprozime^®^) have shown good agronomic performance for *P. ostreatus*, with supplemented mixtures producing mushrooms of higher protein and ash content (Picornell-Buendía et al. 2016b). In addition, quantitative parameters, such as good biological efficiency (BE), high quantity of mushrooms and an excellent unit weight of the fruiting bodies have been achieved with this supplemented SMS when employed to re-grow *P. ostreatus* (Picornell-Buendía et al. 2015). Therefore, spent mushroom substrates supplemented with protein-rich additives can be potentially employed as a cheap base material to grow *P. ostreatus* and simultaneously implement a circular economy based on the integral management of wastes.

## New varieties of cultivated mushrooms to diversify the industry: *Agaricus subrufescens*

**Fig. 2:**
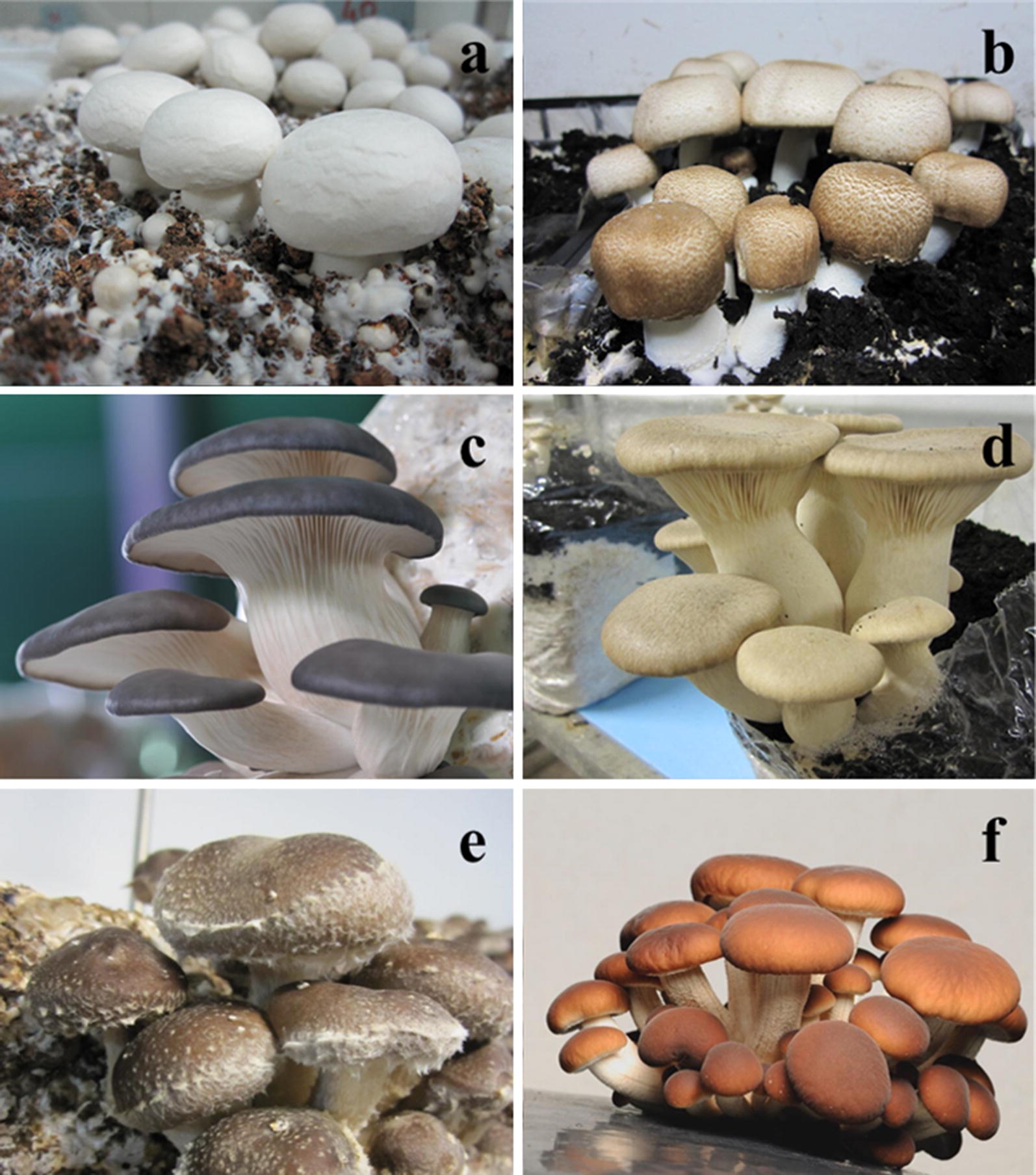
Production of mushrooms on supplemented substrates. **a** *Agaricus bisporus*; **b** *Agaricus subrufescens*; **c** *Pleurotus ostreatus*; **d** *Pleurotus eryngii*; **e** *Lentinula edodes*; **f** *Agrocybe aegerita*

Five different genera constitute 85% of the world’s cultivation of edible mushrooms: *Lentinula edodes* (shiitake), *Agaricus* (mainly *Agaricus bisporus*), *Pleurotus* spp. (5 or 6 species), *Auricularia* and *Flammulina* (Royse et al. 2017). Supplementation has been reported to improve yield and quality of most cultivated species worldwide, including the above cited species (Fig. 2) (Sánchez 2010; Gaitán-Hernández et al. 2014; Liang et al. 2016; Pardo-Giménez et al. 2016, 2018; Xie et al. 2017). Although many other varieties have been successfully cultivated (Carrasco et al. 2018), some of them present commercial limitations due to the reduced yield achieved. Supplementation represents an important strategy to improve the yield and commercial viability of these less widely cultivated varieties.

For example, the practice of supplementation has been an important approach for improving yield in the industrial production of the medicinal mushroom *Agaricus subrufescens* (also known as the sun mushroom) Ellis & Everh (Zied et al. 2018), and therefore is an opportunity to make this new variety commercially viable to diversify the industry. Kopytoswky-Filho et al. (2008) evaluated the effect of supplementation in cultivation of *A. subrufescens*, reporting a significant increase of production with the soybean meal-based commercial supplement Champfood^®^ applied before casing with respect to not supplemented (control) and soybean supplemented. The use of soybean meal and other by-products from soybean transformation, such as commercial formulations, was also reported to have positive effect in the cultivation of sun mushroom by Zied et al. (2011), Wang et al. (2010), Zhou et al. (2010). In addition, waste materials, including agro-industrial waste (provided by peanut and acerola juice) and noble grains, a mix with bran of soybean, corn, and cotton have been proved effective to increase the industrial yield, which highlights materials with high S, Cu, and Mn contents as ideal supplements (Zied et al. 2018).

## Bioinoculants as an alternative or addition to traditional supplementation

Like nutritional supplements it is likely that different bioinoculants can be developed to support mushroom growth at different stages of crop development. Certain microbiota, including bacteria from the genera *Azotobacter*, *Bacillus*, *Paenibacillus* and *Pseudomonas*, described increasing the mycelial growth of cultivated species while showing antagonism against competitive molds, have been reported as candidates for the design of alternative nutritional supplements/biofertilizers (Payapanon et al. 2011; Jadhav et al. 2014; Pratiksha et al. 2017). A good number of bacteria from the genera *Bacillus*, *Pseudomonas* or *Bradyrhizobium* appear to stimulate the mycelial growth of some cultivated species (*A. bisporus*, *A. bitorquis*, *A. subrufescens*, *P. ostreatus* or *P. eryngii*) in compost or in vitro, and others have been described to favor and enhance mushroom fructification in casing, such as members from the genera *Pseudomonas* (Kertesz and Thai 2018). In addition, he colonization of the thermophilic fungus *Mycothermus thermophilus* (*Scytalidium thermophilum*), at the conditioning stage of phase II composting has been described as important for stimulating growth, development and yield of *A. bisporus* and for increasing selectivity of the substrate (Sánchez et al. 2008; Coello-Castillo et al. 2009; Natvig et al. 2015).

Although there are an increasing number of commercial biofertilizers based on bacterial and fungal plant growth promoters (Goswami et al. 2016), to date there are no commercial supplements based on mushroom growth promoting microorganisms available in the market. Next generation sequencing (NGS) is a high-throughput technology that is currently providing a rich source of information about the microorganisms inhabiting the substrates employed in mushroom cultivation and the structure and dynamics of the native microbiota (McGee et al. 2017a, b; Kertesz and Thai 2018; Vieira and Pecchia 2018; our unpublished results). The Omics approach with tools such as metagenomics (to detect and quantify the relative abundance of microorganisms by DNASeq), metatranscriptomics (to study the function and activity through RNASeq) and metabolomics (to characterize metabolites involved with analytical techniques) can set the basis for the development of bioinoculants for the industry while discriminating the most suitable point of application along the crop cycle.

## Overview and perspectives

The agronomic practice of nutritionally supplementing mushroom cultivation is cost effective to improve crop yield and quality; however technical or economical issues limits its globally spread.

The majority of supplements sold commercially are currently based on nitrogen rich compounds, and it is currently unclear whether the use of low-protein supplements based on carbon-rich sources such as cellulose and hemicellulose components improves the performance of the mushroom equally or even more than nitrogen addition. In comparison to protein-rich components, ingredients with high content of carbohydrates, such as agricultural and commercial waste products, are cheaper and readily available in local producing areas (Pardo-Giménez et al. 2016). However, it is noteworthy that the use of commercially available protein-based nutrients is a profitable investment since mushroom yield consistently increases and ultimately also mushroom quality. Besides, the use of nutritional additives is a useful tool to partially recycle the SMS into new growth cycles, in an effort to building a circular economy involving waste management, and to increase the biological efficiency of alternative cultivated species in order to diversify the industry. Although the interactions among mushrooms and their environmental niche has been barely described, microorganisms play an important role in different stages of mushroom cultivation, and therefore future prospects for improving mushroom yield would benefit from deepening the knowledge of the structure, role and dynamics of the mushroom microbiome with the aim of developing supplements based on bioinoculants for mushroom crops.

## Abbreviations

SMS: spent mushroom compost
MGP: mushroom growth promoting
NGS: next generation sequencing
DNASeq: DNA sequencing
RNASeq: RNA sequencing
